# Identification and Validation of a Novel Necroptosis-Related Long Noncoding RNA Prognostic Signature for Lung Adenocarcinoma

**DOI:** 10.1155/2022/9710540

**Published:** 2022-10-25

**Authors:** Cheng Zeng, Huayi Yu, Xiuling Liu, Qian Liu, Jianhua Jin

**Affiliations:** ^1^Department of Oncology, Wujin Hospital Affiliated with Jiangsu University, Changzhou, Jiangsu Province 213017, China; ^2^Department of Oncology, Wujin Clinical College of Xuzhou Medical University, Changzhou, Jiangsu Province 213017, China

## Abstract

**Background:**

Several cancers, including lung adenocarcinoma (LUAD), are caused by genes related to necroptosis. However, it is still unknown how necroptosis-related long noncoding RNAs (lncRNAs) may be involved in LUAD. In order to predict the prognosis of LUAD patients and personalize immunotherapy, this study set out to construct a necroptosis-related lncRNA prognostic signature (NLPS).

**Methods:**

The Cancer Genome Atlas (TCGA) database was used to download the LUAD transcriptome data and the associated clinical data. lncRNAs associated with necroptosis were screened using coexpression analysis. An NLPS was built using univariate and least absolute shrinkage and selection operator (LASSO) Cox regression analyses. The Gene Expression Omnibus (GEO) database's GSE30219 was used to validate the NLPS. The prognostic value of the risk score was assessed using Kaplan-Meier survival, receiver operating characteristic (ROC) Cox regression, multivariate Cox regression, and nomogram analyses. Then, we looked into the differences between the low- and high-risk groups in the tumor immune microenvironment, immunotherapy response, and half-maximal inhibitory concentration (IC50).

**Results:**

The 14 lncRNAs in a novel NLPS were created. With further validation in the GSE30219 dataset, the risk score according to the NLPS was an independent prognostic indicator for LUAD patients. Patients with better overall survival (OS) in the low-risk group, who were characterized by increased immune cell infiltration, tumor mutational burden (TMB), and immunophenoscore (IPS), may have hot tumors and higher immunotherapy response rates. In addition, the risk score was also closely linked to sensitivity to various anticancer medications.

**Conclusions:**

We constructed a novel NLPS that could predict OS and sensitivity to immunotherapy in LUAD patients.

## 1. Introduction

According to estimates, 2.2 million new instances of cancer and 1.8 million cancer-related deaths will be attributable to lung cancer in 2020 [1]. Clinically, lung adenocarcinoma (LUAD) accounts for 53% of non-small cell lung cancer (NSCLC) cases in China, while NSCLC accounts for 85% of all instances of lung cancer [2]. Even though the development of targeted therapy and immunotherapy has revolutionized the treatment of NSCLC, patients with distant metastasis show a 5-year survival rate of about 7% [3], and only 20% of NSCLC patients exhibit a significant response to targeted therapy and immunotherapy [4]. Therefore, it is critical to construct a novel robust prognostic signature to screen out LUAD patients most likely to benefit from immunotherapy.

Necroptosis is a unique kind of controlled cell death that excludes the involvement of proteins from the caspase family [5]. According to growing research, necroptosis has been connected to several diseases, including inflammation, myocardial infarction, neurodegenerative disorders, autoimmune diseases, infectious diseases, and cancers [6, 7]. Necroptosis plays a double-edged sword in the incidence and progression of tumors. For instance, necroptosis can promote tumor cell proliferation, invasion, and metastasis and is associated with a poor prognosis in many malignancies [8–10]. Interestingly, necroptosis can enhance the antitumor immune response by activating CD8+ T cells, thereby inhibiting tumorigenesis and development [7, 11]. A study also found that necroptosis promotes the growth of pancreatic tumors by creating an immunosuppressive tumor microenvironment caused by suppressive macrophages [9]. However, it is still unclear what the potential underlying mechanisms and prognostic significance of necroptosis in LUAD are.

Long noncoding RNAs (lncRNAs) are RNAs longer than 200 nucleotides that do not code for proteins. lncRNAs have the ability to bind to proteins, DNA, and RNA to control several aspects of gene expression, including transcription, posttranscriptional processing, RNA metabolism, translation, and posttranslational modification [12]. Numerous studies have shown the significance of lncRNAs in programmed cell death, including apoptosis, ferroptosis, autophagy, and necroptosis [13]. For instance, it has been observed that the miR-675, produced from the lncRNA H19, increases p-MLKL and RIP3 while decreasing the expression of FADD, leading to the necroptosis of liver cancer cells [14]. Zhao et al. also used necroptosis-related lncRNAs to forecast the prognoses of gastric cancer patients [15]. However, the biological significance of necroptosis-related lncRNAs in LUAD and their predictive usefulness have not yet been determined.

The TCGA database was used to obtain the LUAD patients' lncRNA and mRNA expression patterns and clinical data. Then, using univariate Cox regression and LASSO Cox regression analyses, we created an NLPS using the TCGA cohort. The NLPS was verified using GSE30219, retrieved from the GEO database. Next, discrepancies in possible signaling pathways, tumor microenvironment (TME), TMB, IPS, and half-maximal inhibitory concentration (IC50) values between the low- and high-risk groups were identified according to the NLPS. We anticipate that our findings will offer a fresh viewpoint for gauging the LUAD patients' prognosis and creating customized immunotherapy.

## 2. Materials and Methods

### 2.1. Data Collection

From the TCGA database (https://portal.gdc.cancer.gov/), we downloaded the expression profile data, mutation data, and associated clinical data of LUAD patients. The associated clinical information and sample expression data were then combined. Patients without information on overall survival (OS) were excluded. The GEO database was used to retrieve GSE30219, which used the GPL570 platform and contained clinical and transcriptomic data. Using the R package sva, the TCGA and GEO expression matrixes were adjusted to the same level. The samples from the TCGA dataset were used as the training cohort, and the samples from GSE30219, retrieved from the GEO database, were used as the validation cohort. From earlier reviews, 67 genes associated with necroptosis were obtained (Table S1) [15, 16].

### 2.2. Construction of an NLPS for LUAD

The Pearson correlation coefficient method was used to screen necroptosis-related lncRNAs with |*R*| > 0.4 and *P* < 0.001. A univariate Cox regression analysis was used to find the necroptosis-related lncRNAs associated with OS in LUAD patients. The R package glmnet was employed to conduct LASSO Cox regression analysis to construct a predictive signature for necroptosis [17]. The best signature with the lowest Akaike information criterion (AIC) value was chosen to reduce the danger of overfitting [18]. The expression levels and regression coefficients of lncRNAs were utilized to determine each LUAD patient's risk score [19]. Risk score is calculated as follows: *β* lncRNA1 × exp lncRNA1 + *β* lncRNA2 × exp lncRNA2 + ⋯.+*β* lncRNAn × exp lncRNAn. The patients were divided into the low- and high-risk groups separately according to the median risk score calculated in the training cohort. [20].

### 2.3. Survival Analysis and Receiver Operating Characteristic (ROC) Curve Plotting

The R package survminer and survival was subjected to perform a Kaplan-Meier survival analysis to compare OS between the low- and high-risk groups in the training cohort and the validation cohort [21]. For LUAD patients, we further conducted subgroup OS analysis based on clinicopathological characteristics. The R package survivalROC was used to plot ROC curves and determine the area under the curve (AUC) values [22].

### 2.4. Independent Prognostic and Nomogram Analysis

In the training and validation cohorts, univariate and multivariate Cox regression analyses were carried out to determine if the risk score based on the NLPS could be considered an independent prognostic factor for LUAD patients. Age, sex, clinical stage, T stage, M stage, N stage, and risk score were the factors. A nomogram was created based on the risk score of the NLPS and clinical pathological factors to predict the 1-year, 3-year, and 5-year survival of LUAD patients [23]. Calibration curves were produced to assess the nomograms' effectiveness in predicting OS.

### 2.5. Principal Component Analysis (PCA) and Gene Set Enrichment Analysis (GSEA)

PCA was performed to examine the distribution of patients with various risk scores using the R package stats [24]. The signaling pathways and biological processes connected to the low- and high-risk groups were examined using GSEA software (version 4.1.0) [25]. The c2.cp.kegg.v7.4.symbols.gmt was selected for annotated gene sets. The normalized enrichment score (NES), as determined by the Affymetrix chip platform, was computed after 1,000 permutations. Gene sets were significantly differentially enriched with normal *P* < 0.05 and false discovery rate (FDR) *P* < 0.25.

### 2.6. Tumor Immune Microenvironment Analysis

The ESTIMATE method, which incorporates stromal, immune, and ESTIMATE scores, was utilized to investigate the differences in the immunological microenvironment between the low- and high-risk groups. XCELL, TIMER, QUANTISEQ, MCPCOUNTER, EPIC, CIBERSORT-ABS, and CIBERSORT algorithms were used in the Spearman correlation analysis of the risk score based on the NLPS and immune cells [15]. Single-sample GSEA (ssGSEA) was used to determine the scores of infiltrating immune cells and immune-related pathways using the R package gsva [26].

### 2.7. Immunotherapy Response Analysis

We initially compared the levels of gene expression for immune checkpoint-related proteins across the low- and high-risk groups to investigate the significance of the NLPS in predicting the outcome of immunotherapy. According to studies, the TMB and IPS can indicate how well patients will respond to immunotherapy, and those who have higher TMB or IPS may benefit from immune checkpoint inhibitors [19, 27]. For each patient with LUAD, we determined the TMB and coupled a survival analysis with a risk score. In addition, we did a differential analysis between the low- and high-risk groups using the IPS scores of LUAD patients that we acquired from The Cancer Immunome Atlas (https://tcia.at/).

### 2.8. IC50 Analysis

The R package pRRophetic was used to compare the IC50 values of LUAD patients in the low- and high-risk groups to assess the NLPS in the prospective therapeutic application of LUAD treatment [28]. *P* < 0.05 was considered statistically significant.

### 2.9. Statistical Analysis

For the statistical analysis, R software (version 4.1.2) was used. Clinicopathological traits, immunological state, TMB, IPS score, and IC50 values were compared between groups using the Wilcoxon test. The survival of several groups was compared using Kaplan-Meier curves. The examination of independent prognostic variables included both univariate and multivariate Cox regression. The prediction ability of the NLPS was assessed using ROC curves. *P* < 0.05 was considered statistically significant. ^∗^*P* < 0.05, ^∗∗^*P* < 0.01, and ^∗∗∗^*P* < 0.001.

## 3. Results

### 3.1. Identification of an NLPS in LUAD

**Figure 1:**
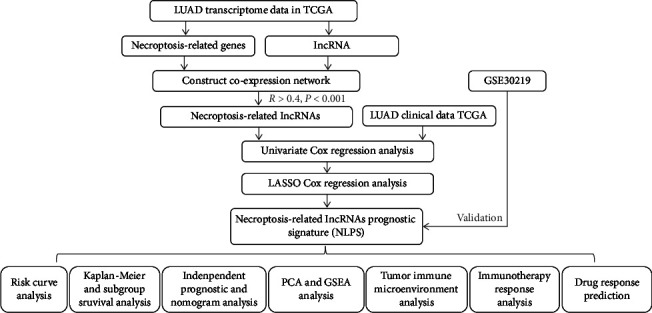
Schematic diagram of the study design.

**Figure 2:**
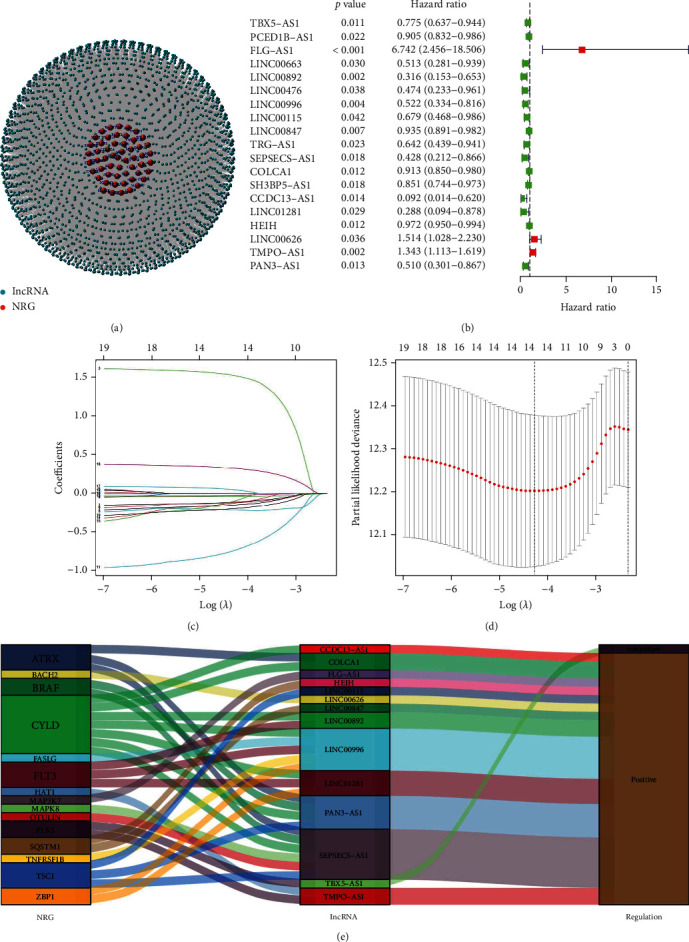
Identification of a necroptosis-related lncRNA prognostic signature (NLPS) in LUAD. (a) The coexpression network map of necroptosis-related genes and long noncoding RNAs with |*R*| > 0.4 and *P* < 0.001 as the screening criteria. (b) Prognostic forest map of necroptosis-related lncRNAs by univariate Cox regression analysis. (c, d) LASSO Cox regression analysis of 19 prognostic necroptosis-related lncRNAs. (e) Sankey diagram of necroptosis-related genes and lncRNAs.

A schematic of the research plan is shown in Figure 1. Patients from the TCGA dataset were utilized as the training cohort. The coexpression analysis of 67 necroptosis-related genes with |*R*| > 0.4 and *P* < 0.001 resulted in the identification of 2154 necroptosis-related lncRNAs in total (Figure 2(a), Table S2). 19 lncRNAs associated with necroptosis were shown to be significantly correlated with the OS in LUAD patients, according to a univariate Cox regression analysis (Figure 2(b)). LASSO Cox regression analysis was utilized to limit the possibility of overfitting of necroptosis-related prognostic lncRNAs, and 14 of 19 necroptosis-related lncRNAs were selected to create the NLPS (Figures 2(c) and 2(d)). The risk score of the NLPS was calculated as follows: risk score = (−0.11275 × TBX5 − AS1) + (1.53372 × FLG − AS1) + (−0.21258 × LINC00892) + (−0.15068 × LINC00996) + (−0.12119 × LINC00115) + (−0.04065 × LINC00847) + (−0.74594 × SEPSECS − AS1) + (−0.02410 × COLCA1) + (−0.12741 × CCDC13 − AS1) + (−0.10122 × LINC01281) + (−0.00085 × HEIH) + (0.04878 × LINC00626) + (0.32701 × TMPO − AS1) + (−0.19790 × PAN3 − AS1). The Sankey diagram showed that PLK1 and TBX5-AS1 expression levels were negatively connected, in contrast to the other genes and lncRNAs, whose expression levels were all positively associated (Figure 2(e)).

### 3.2. Evaluation and Validation of the NLPS

**Figure 3:**
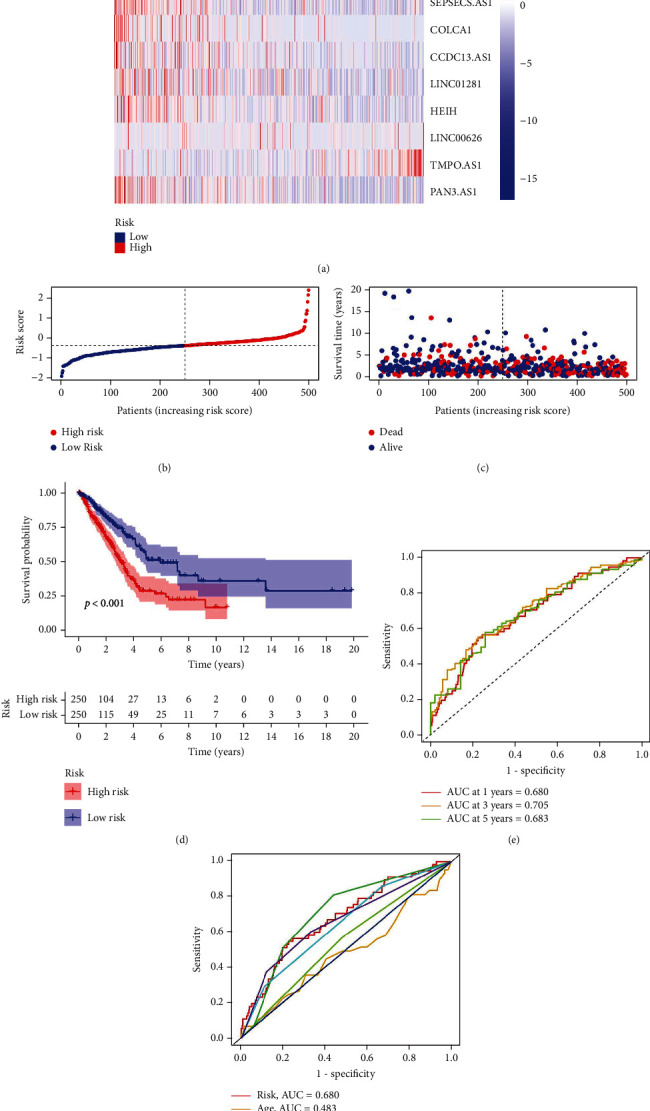
Verification of the NLPS in the TCGA cohort. (a) Heat map of 13 necroptosis-related lncRNA expression levels. (b) Distribution of risk scores. (c) Scatterplot of survival status. (d) Kaplan-Meier survival curves of the low- and high-risk groups stratified by the NLPS. (e) 1-, 2-, and 3-year ROC curves. (f) 1-year ROC curves of the risk score and clinical characteristics.

The median risk score was employed to divide LUAD patients into the low- and high-risk groups. Samples from the TCGA dataset were used as the training cohort. The heat map displays the expression levels of 14 lncRNAs linked to necroptosis (Figure 3(a)). The risk curves and scatter plots demonstrated that the training cohort's LUAD patients had shorter survival times the higher their risk scores were (Figures 3(b) and 3(c)). The training cohort's Kaplan-Meier survival analysis showed that the high-risk group's LUAD patients' OS was considerably shorter than that of the low-risk group's LUAD patients (Figure 3(d)). We conducted a ROC analysis and estimated the AUC value of the risk score based on the NLPS to assess the prediction performance of the NLPS. The training cohort's 1-, 3-, and 5-year AUC values were 0.680, 0.705, and 0.683, respectively (Figure 3(e)). Additionally, we discovered that the risk score's 1-year AUC value exceeded 0.60 and was higher than the training cohort's AUC values for age and sex (Figure 3(f)).

**Figure 4:**
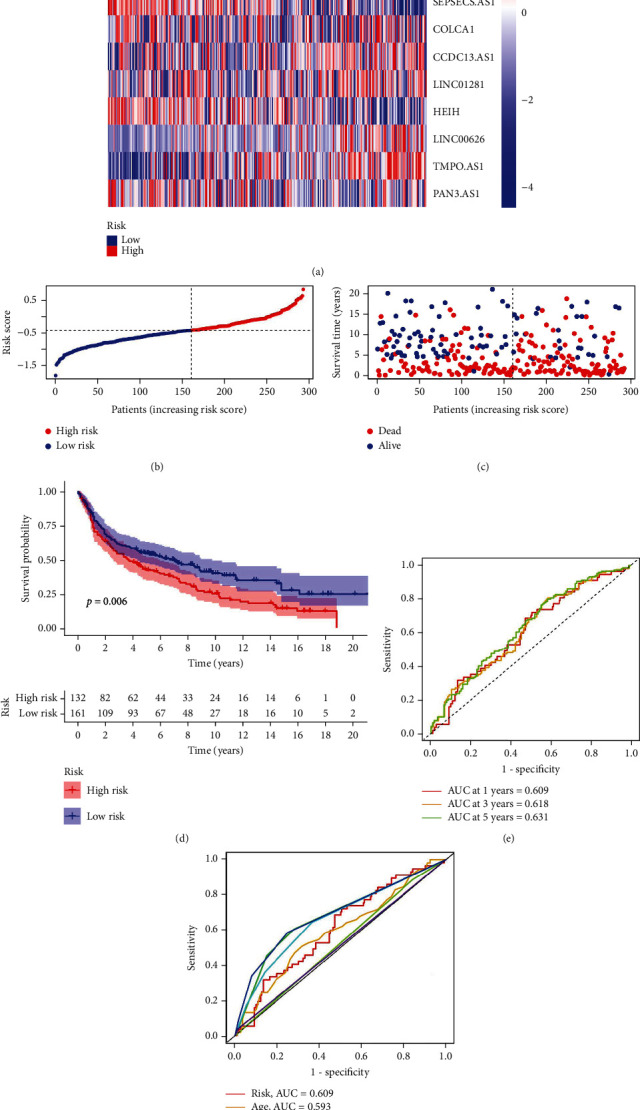
Validation of the NLPS in the GEO cohort. (a) Heat map of 13 necroptosis-related lncRNA expression levels in GSE30219. (b) Distribution of risk scores. (c) Scatterplot of the survival status. (d) Kaplan-Meier survival curves of the low- and high-risk groups stratified by the NLPS. (e) 1-, 2-, and 3-year ROC curves. (f) 1-year ROC curves of the risk score and clinical characteristics.

Samples from GSE30219, retrieved from the GEO database, were utilized as the validation cohort to confirm the NLPS's applicability. We ran the same study in the validation cohort and got similar results. The validation cohort's 14 necroptosis-related lncRNA expression patterns are displayed in Figure 4(a). The distributions of the risk score and survival time in the validation cohort are shown in Figures 4(b) and 4(c), respectively. Patients with greater risk ratings had shorter OS than patients with lower risk scores, as seen in Figure 4(d). The validation cohort's 1-, 3-, and 5-year AUC values were 0.609, 0.618, and 0.631, respectively (Figure 4(e)). In the validation cohort, the risk score's 1-year AUC value was higher than those of age, sex, and M stage (Figure 4(f)).

### 3.3. Subgroup Analysis of the NLPS

**Figure 5:**
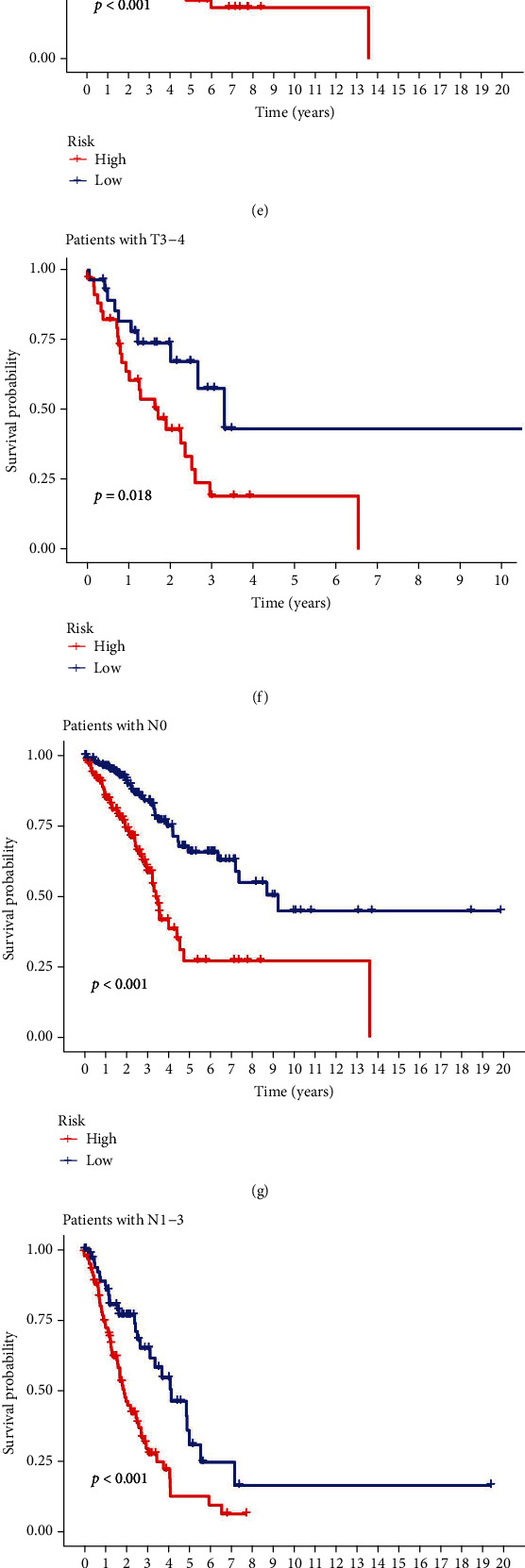
Subgroup analysis of the NLPS: (a) Kaplan-Meier survival analysis for age > 65 years, (b) age ≤ 65 years, (c) female, (d) male, (e) T1-2, (f) T3-4, (g) N0, (h) N1-3, (i) M0, (j) M1, (k) stage I–II, and (l) stage III–IV between the low- and high-risk groups.

We used subgroup analysis to examine if the NLPS was connected to the clinical characteristics of LUAD patients. Age (>65 years or 65 years), sex (male or female), T stage (T1-2 or T3-4), N stage (N0 or N1-3), M stage (M0 or M1), and clinical stage (stage I–II or stage III–IV) were used to separate the groupings. When sorted by age, sex, T stage, N stage, M stage, and clinical stage, we discovered that the OS of high-risk LUAD patients was significantly shorter than that of low-risk LUAD patients (all *P* < 0.05) (Figure 5).

### 3.4. The Risk Score Is an Independent Prognostic Factor

**Figure 6:**
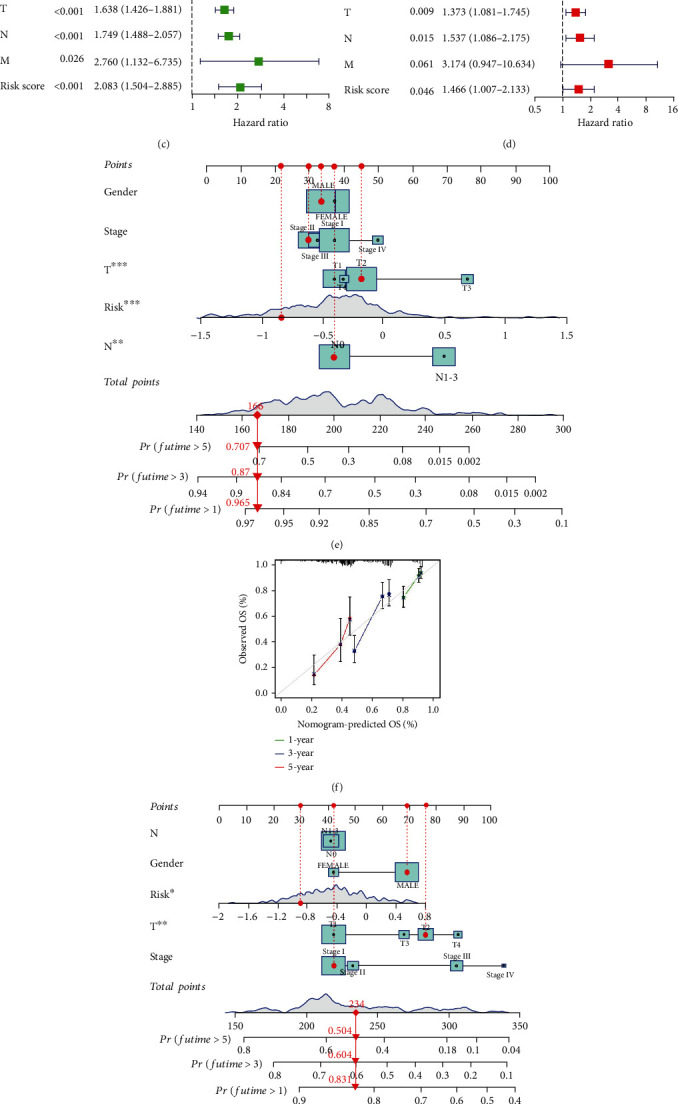
Independent prognostic and nomogram analysis. (a) Univariate and (b) multivariate Cox regression analyses of clinical features and risk scores with OS in the TCGA cohort and (c, d) GEO cohort. (e) The nomogram combining gender, clinical stage, T stage, N stage, and risk score for predicting LUAD patient OS at 1, 3, and 5 years in the TCGA cohort and (g) GEO cohort. (f) The nomogram calibration curves for 1-, 3-, and 5-year OS in the TCGA cohort and (h) GEO cohort.

We performed univariate and multivariate Cox regression analyses in the training and validation cohorts to see if the risk score could be an independent prognostic factor for LUAD patients. Univariate Cox regression analysis of the training cohort showed that clinical stage (HR = 1.577, 1.348-1.845, *P* < 0.001), T stage (HR = 1.579, 1.296-1.923, *P* < 0.001), M stage (HR = 1.843, 1.038-3.272, *P* = 0.037), N stage (HR = 1.706, 1.405-2.072, *P* < 0.001), and risk score (HR = 3.506, 2.558-4.807, *P* < 0.001) predicted worse OS (Figure 6(a)). Multivariate Cox regression analysis verified that the risk score was an independent prognostic factor in LUAD patients (HR = 3.128, 2.211-4.424, *P* < 0.001) (Figure 6(b)). Further evidence that the risk score was a standalone predictive factor was provided by the validation cohort's results (HR = 1.466, 1.007-2.133, *P* = 0.046) (Figures 6(c) and 6(d)). Additionally, we combined the variables of sex, clinical stage, T stage, N stage, and risk score to create a nomogram that predicted the survival rates of the training cohort and validation cohort at 1, 3, and 5 years (Figures 6(e) and 6(g)). The 1-, 3-, and 5-year calibration curves demonstrated that the projected OS from the nomogram agreed with the actual OS (Figures 6(f) and 6(h)).

### 3.5. PCA and GSEA

**Figure 7:**
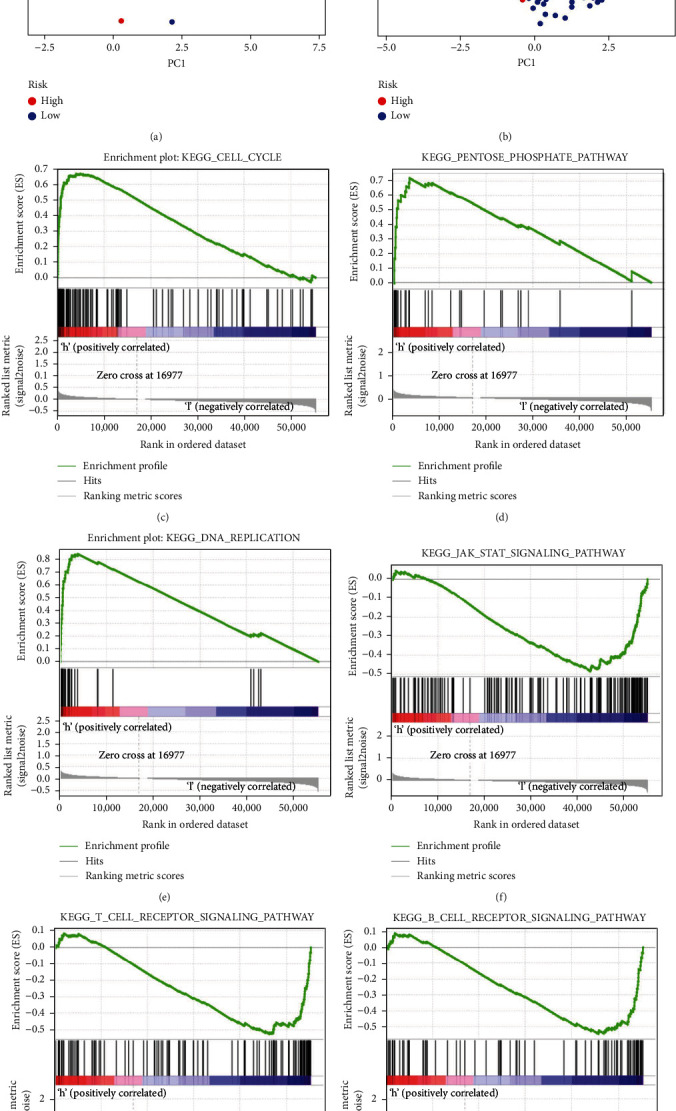
Principal component analysis (PCA) and gene set enrichment analysis (GSEA). (a) PCA of the low- and high-risk groups in the TCGA cohort and (b) GEO cohort. (c) The cell cycle, (d) pentose phosphate pathway, and (e) DNA replication were activated in the high-risk group. (f) The JAK/STAT signaling pathway, (g) T cell receptor signaling pathway, and (h) B cell receptor signaling pathway were activated in the low-risk group.

Based on the NLPS, PCA was used to visualize the patient distribution (Figures 7(a) and 7(b)). The KEGG enrichment analysis was carried out using GSEA software to investigate the variations in potential biological activities between the two subgroups. We found that the high-risk group was related to the cell cycle, pentose phosphate pathway, and DNA replication. In contrast, the low-risk group was linked to the JAK/STA, T cell receptor, and B cell receptor signaling pathways (Figures 7(c)–7(h)).

### 3.6. Tumor Immune Microenvironment Analysis

**Figure 8:**
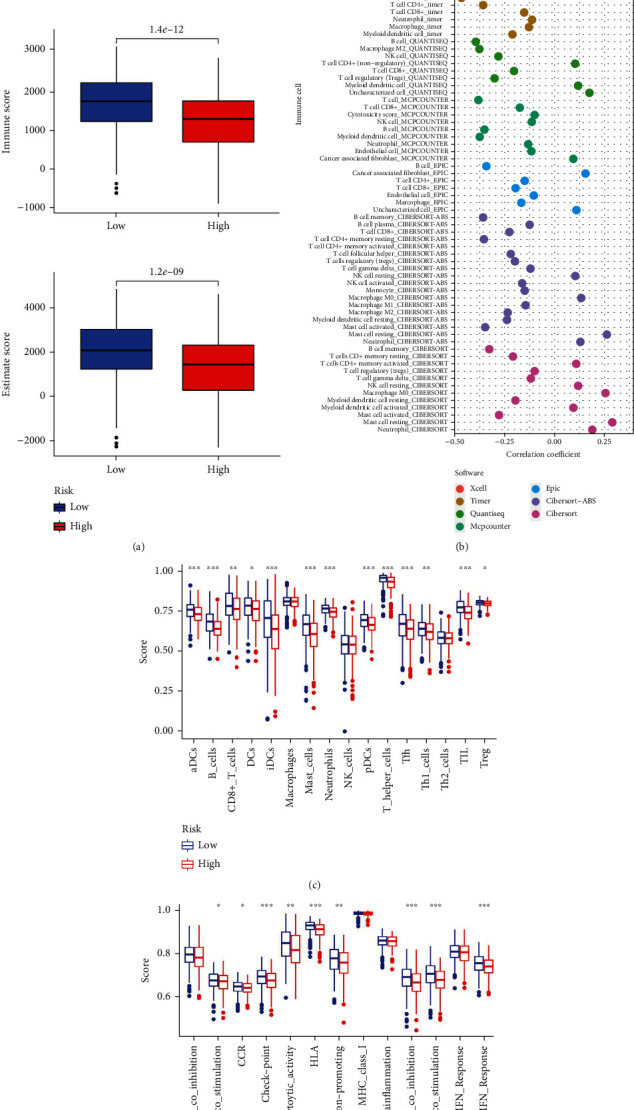
Tumor immune microenvironment analysis. (a) Immune microenvironment analysis between the low- and high-risk groups by ESTIMATE. (b) Spearman correlation analysis of the risk score and immune cells based on the XCELL, TIMER, QUANTISEQ, MCPCOUNTER, EPIC, CIBERSORT-ABS, and CIBERSORT algorithms. (c) Immune cells and (d) immune-related function analyses between the low- and high-risk groups by ssGSEA. ^∗^*P* < 0.05, ^∗∗^*P* < 0.01, and ^∗∗∗^*P* < 0.001.

ESTIMATE analysis was carried out to investigate variations in the tumor microenvironment between the low- and high-risk groups. We discovered that patients in the low-risk group had poor tumor purity because their stromal scores, immunological scores, and ESTIMATE scores were considerably higher in the low-risk group than in the high-risk group (Figure 8(a)). A bubble chart created using seven different algorithms later revealed that the risk score was negatively correlated with B cells, T cell CD8+, T cell CD4+, and cancer-associated fibroblast, while positively correlated with T cell CD4+ Th1, T cell CD4+ Th2, NK cell resting, and mast cell resting (Figure 8(b), Table S3). These findings imply that patients in the low-risk group may have had hot tumors with more immune cell infiltration. Additionally, the low- and high-risk groups' immune cell infiltration and immune-related pathways were compared using ssGSEA. We discovered that activated dendritic cells (aDCs), B cells, T cell CD8+, dendritic cells (iDCs), immature dendritic cells (iDCs), mast cells, neutrophils, plasmacytoid dendritic cells (pDCs), T helper cells, follicular helper T cell (Tfh), Th1 cells, tumor-infiltrating lymphocyte (TIL), and regulatory T cell (Treg) had higher enrichment scores in the low-risk group than in high-risk group (Figure 8(c)). Immune function analysis suggested that APC costimulation, CCR, checkpoint, cytolytic activity, HLA, inflammation-promoting, T cell coinhibition, T cell costimulation, and type II IFN response pathways exhibited higher activity in the low-risk group than in the high-risk group (Figure 8(d)).

### 3.7. Immunotherapy Response Analysis

**Figure 9:**
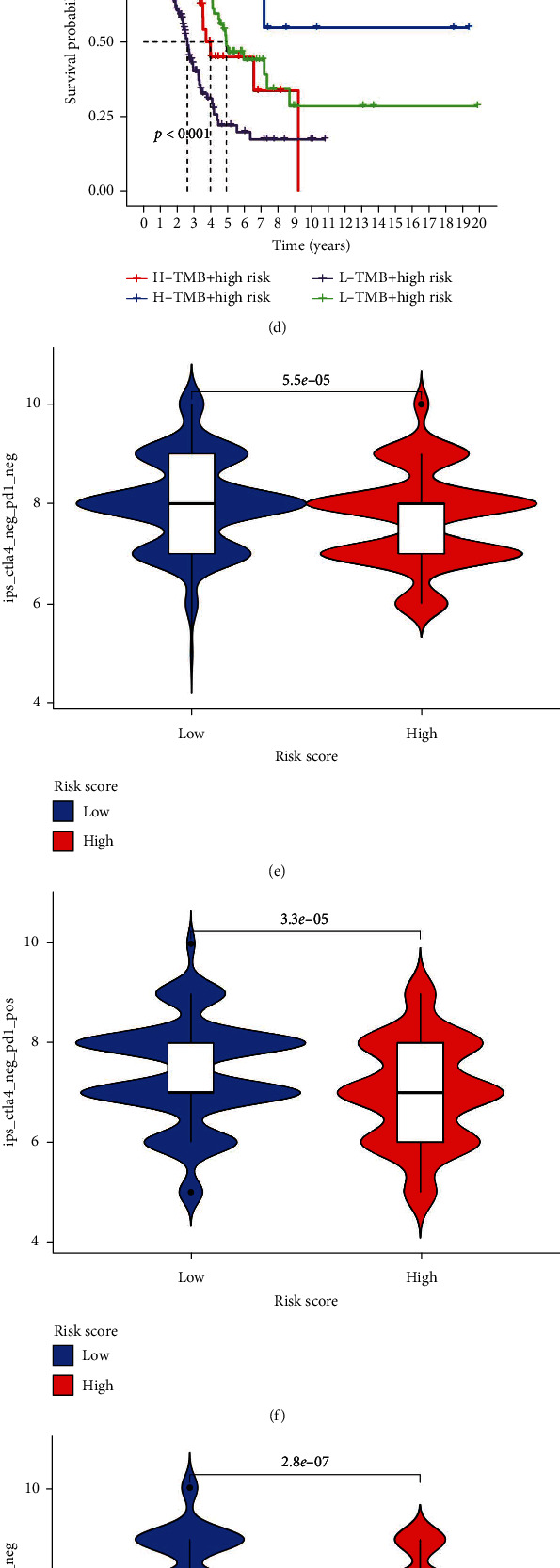
Immunotherapy response analysis. (a) Immune checkpoint-related gene expression level analysis between the low- and high-risk groups, which were classified by the NLPS. (b) Tumor mutational burden analysis between the low- and high-risk groups. (c) Survival analysis of LUAD patients with high and low tumor mutational burden. (d) Survival analysis combined with tumor mutational burden status and risk score. (e) Ips_ctla4_neg_pd1_neg, (f) ips_ctla4_neg_pd1_pos, (g) ips_ctla4_pos_pd1_neg, and (h) ips_ctla4_pos_pd1_pos score analyses between the two subgroups.

Since the expression levels of immune checkpoint-related genes have become markers for treatment choice in LUAD patients, we examined the expression levels of these genes in the low- and high-risk groups. The findings revealed that the expression levels of most immune checkpoint-related genes, including CTLA4, PDCD1, CD274, PD-L1, LAG3, and HAVCR2 (TIM3), were greater in the low-risk group than in the high-risk group (Figure 9(a)). According to studies, the TMB and IPS are reliable predictors of immunotherapeutic response [19, 27]. According to our findings, individuals with LUAD in the low-risk group had a higher TMB than those in the high-risk group (Figure 9(b)), which was consistent with their superior OS (Figure 9(c)). Additionally, a survival analysis that included the TMB and risk score revealed that the risk score decreased the higher TMB group's better prognosis (Figure 9(d)). Furthermore, we examined the variations between two subgroups after downloading the IPS score from the TCIA database. Our findings demonstrated that LUAD patients in the low-risk group had higher IPS (Ips_ctla4_neg_pd1_neg, ips_ctla4_neg_pd1_pos, ips_ctla4_pos_pd1_neg, and ips_ctla4_pos_pd1_pos scores) than those in the high-risk group (Figures 9(e)–9(h)), indicating that these individuals' immunogenicity was greater. In summary, the better outcomes of patients at low risk, according to the NLPS, occur due to their better response to immune checkpoint inhibitor treatment.

### 3.8. Analysis of Drug Sensitivity

**Figure 10:**
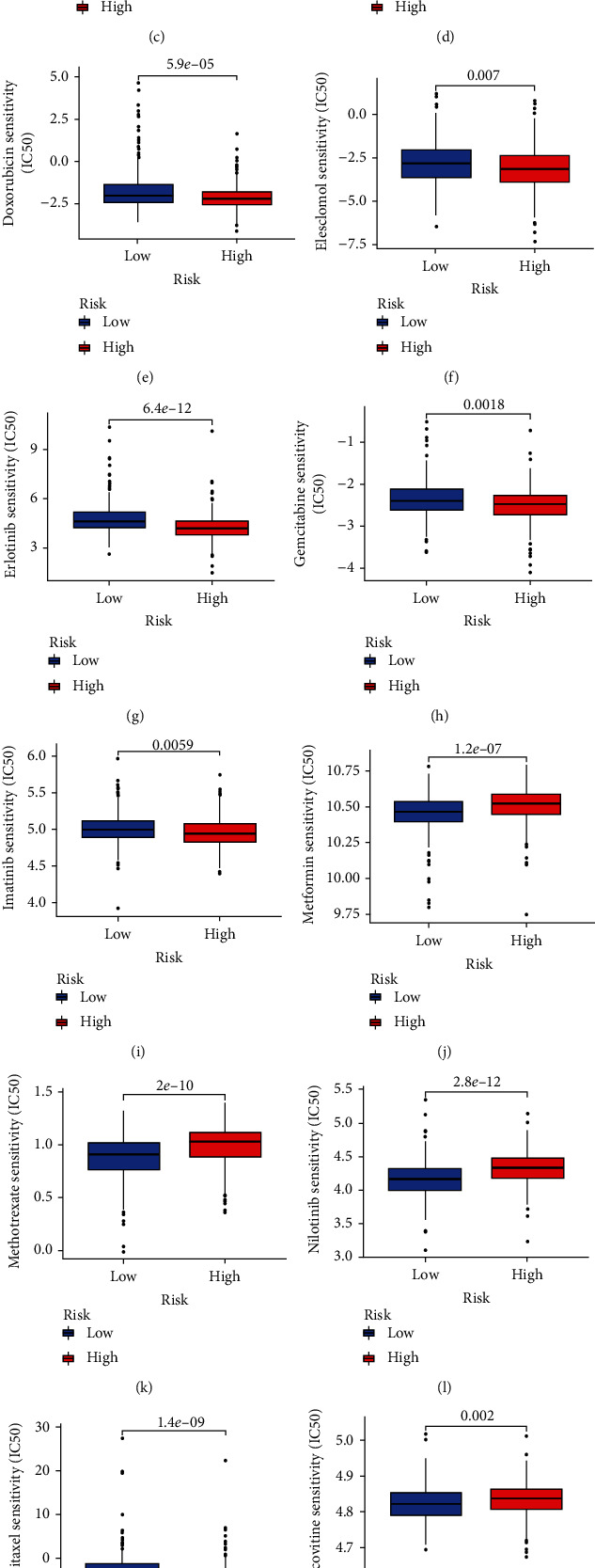
Drug sensitivity prediction. (a) IC50 analysis of axitinib, (b) bortezomib, (c) dasatinib, (d) docetaxel, (e) doxorubicin, (f) elesclomol, (g) erlotinib, (h) gemcitabine, (i) imatinib, (j) metformin, (k) methotrexate, (l) nilotinib, (m) paclitaxel, (n) roscovitine, (o) sorafenib, and (p) vinblastine in the low- and high-risk groups, which were classified by the NLPS.

We evaluated the variations in the IC50 values of typical antitumor drugs in the low- and high-risk groups to investigate whether the NLPS can predict the responsiveness of LUAD patients to antitumor drugs. We discovered that LUAD patients in the low-risk group were more sensitive to axitinib, metformin, methotrexate, nilotinib, roscovitine, and vinblastine but less sensitive to bortezomib, dasatinib, docetaxel, doxorubicin, elesclomol, erlotinib, gemcitabine, imatinib, paclitaxel, and sorafenib (Figure 10). These findings suggested that our NLPS could be a valuable predictor of drug sensitivity.

## 4. Discussion

In our work, coexpression analysis was utilized to obtained 2154 lncRNAs related to necroptosis in the training cohort. Then, an NLPS composed of 14 lncRNAs was created using LASSO and univariate Cox regression analyses. We calculated the risk score for each patient and then separated the patients into low- and high-risk groups according to the median risk score. The OS of patients in the low-risk group was higher than those in the high-risk group, demonstrating that the NLPS may predict LUAD patient prognosis. We further verified the subgroup survival analysis's prediction efficacy. According to the NLPS, the risk score was also discovered to be an independent prognostic indicator for LUAD patients. A nomogram that combined the risk score and clinicopathological traits successfully accurately forecasted the 1-, 3-, and 5-year survival rates in LUAD patients. GSE30219 was employed as the validation cohort to confirm the validity of the NLPS. We calculated the risk score for each patient in the GSE30219 cohort using the risk score formula and divided patients into low- and high-risk groups by the median risk score calculated in the TCGA-LUAD cohort after adjusting the TCGA-LUAD and GSE30219 expression profile data to the same level. We carried out the same research on the GSE30219 cohort, and incredibly, we saw findings that were quite similar, further demonstrating the NLPS's remarkable predictive power.

Among the 14 necroptosis-related lncRNAs in the NLPS, SEPSECS-AS1, CCDC13-AS1, and LINC00626 were first identified. Studies have shown that TBX5-AS1, a novel prognostic marker [29], is expressed at low levels in NSCLC. It can inhibit tumor cell proliferation, invasion, and migration and promote apoptosis through the PI3K/AKT signaling pathway [29]. A multicenter study showed that FLG-AS1 could be used to forecast the pathological complete response rates to neoadjuvant chemotherapy and radiation therapy for esophageal squamous cell carcinoma [30]. However, there is no relevant research on FLG-AS1 in LUAD. The previous study demonstrated that LINC00892 and LINC00996 are immune-related lncRNAs and could be used to forecast the response rate to immunotherapy [31, 32], and LINC00996 might be a potential LUAD therapeutic target [32]. Wu et al. demonstrated that LINC00115 promotes tumor progression through the miR-607/ITGB1 pathway [33]. Li et al. demonstrated that E2F1-induced LINC00847 promoted NSCLC cell proliferation, invasion, and migration by targeting the miR-147a/IFITM1 axis [34]. Zheng et al. constructed a LUAD prognostic signature, and this signature included COLCA1, TMPO-AS1, and TBX5-AS1 [35], which were also included in our prognostic signature. Ye et al. demonstrated that LINC01281 enhanced T cells' capacity to migrate to tumor cells using a T cell chemotaxis assay [36]. In addition, LINC01281 is considered a protective factor for laryngeal cancer [37]. Ping et al. identified a ferroptosis-related lncRNA prognostic signature that included PAN3-AS1 and found it associated with a favorable prognosis in cancer patients [38]. HEIH is highly expressed in several tumor types; can promote tumor cell proliferation, migration, invasion, and drug resistance; and is related to a poor prognosis [39].

PCA was initially carried out in the TCGA-LUAD cohort and the GSE30219 cohort better to examine the role of NLPS in biological function. According to the NLPS, we discovered that LUAD patients could be distinguished into two categories. Afterward, GSEA was conducted to investigate biological function variations between the low- and high-risk groups. We discovered that the low-risk group had active JAK/STAT, T cell receptor, and B cell receptor signaling pathways. The JAK/STAT signaling pathway is thought to be a viable approach for tumor immunotherapy because it plays a crucial role in immune regulatory processes [40]. The body's immune system consists of T cells and B cells, both of which can be activated to fight tumors. Patients in the low-risk group had better OS, which may be related to an active tumor immune microenvironment. As a result, we investigated immunological infiltration between the two categories in more detail. According to ESTIMATE analysis, individuals in the low-risk group had higher stromal, immunological, and ESTIMATE scores, indicating that these patients had poor tumor purity. Consistently, the risk score was inversely related to most immune cells, including CD8+ T cells, CD4+ T cells, and B cells. ssGSEA further demonstrated that patients in the low-risk group had more immune cell infiltration and active immune function. Studies have shown that type II IFN, which is connected to the JAK/STAT pathway, can increase the cytotoxicity of CD8+ T cells and NK cells and accelerate tumor cell senescence and apoptosis [40–43]. Our research revealed that the low-risk group of LUAD patients had a more robust type II IFN response, indicating a link between low-risk patients' improved clinical outcomes and an active antitumor immune response. Subsequently, we performed a response analysis for immunotherapy. We discovered that immune checkpoint-related genes were expressed at greater levels in LUAD patients in the low-risk group, such as CTLA4, PDCD1 (PD1), CD274 (PD-L1), LAG3, and HAVCR2 (TIM3). According to studies, the TMB and IPS are reliable predictors of immunotherapeutic response [19, 27]. Higher TMB and IPS patients frequently had improved OS. Our findings indicated that the low-risk group had a greater TMB and IPS, implying that patients with LUAD who are in the low-risk group may benefit more from immunotherapy. Studies have shown that tumors that respond to immune checkpoint inhibitors exhibit higher levels of immune infiltration and IFN, known as “hot tumors.” In contrast, “cold tumors” have lower levels of immune infiltration and are less responsive to immune checkpoint inhibitors [44]. Therefore, we defined low-risk patients as having “hot tumors,” a higher response rate to immunotherapy, and a better prognosis, while high-risk patients were defined as having “cold tumors,” a lower response rate to immunotherapy, and a poorer prognosis, which further demonstrated the superiority of our NLPS.

The efficacy of antitumor drugs is closely related to the drug sensitivity of patients, and the use of drugs to which patients are susceptible will significantly enhance the therapeutic impact of anticancer medications. Therefore, we further analyzed the IC50 values of antitumor drugs in two subgroups. The results showed that axitinib, metformin, methotrexate, nilotinib, roscovitine, and vinblastine were the ideal choices for LUAD patients in the low-risk group, while bortezomib, dasatinib, docetaxel, doxorubicin, elesclomol, erlotinib, gemcitabine, imatinib, paclitaxel, and sorafenib may work better for LUAD patients in the high-risk group. Our findings may provide prospective options for the clinical treatment of LUAD patients.

However, our studies have several shortcomings. First, this was a retrospective study based on public databases. We constructed the NLPS using the TCGA-LUAD cohort and validated it in the GSE30129 cohort. However, large-scale prospective investigations are still needed in the future to validate our prognostic signature. In addition, *in vivo* and *in vitro* experiments are required to comprehend the probable mechanism of the risk score based on the NLPS.

## 5. Conclusion

Our study constructed a novel NLPS that integrates 14 lncRNAs (TBX5-AS1, FLG-AS1, LINC00892, INC00996, LINC00115, LINC00847, SEPSECS-AS1, COLCA1, CCDC13-AS1, LINC01281, HEIH, LINC00626, TMPO-AS1, and PAN3-AS1) that can predict prognosis. Moreover, patients in the low-risk group had a better prognosis, which may be related to the benefit of immunotherapy. However, more clinical experiments are needed in the future to verify the performance of the NLPS.

## Data Availability

The TCGA-LUAD dataset and GSE30219 dataset are available by contacting the author.

## Abbreviations

LUAD: Lung adenocarcinoma
NLPS: Necroptosis-related lncRNA prognostic signature
TCGA: The Cancer Genome Atlas
KEGG: Kyoto Encyclopedia of Genes and Genomes
FDR: False discovery rate
LASSO: Least absolute shrinkage and selection operator
AIC: Akaike information criterion
ROC: Receiver operating characteristic
AUCs: Area under the curve
PCA: Principal component analysis
IC50: Half inhibitory centration
ssGSEA: Single-sample gene set enrichment analysis.

## Supplementary Materials

Supplementary Materials Table S1: a list of 67 necroptosis-related genes. Table S2: a list of 2154 necroptosis-related lncRNAs. Table S3: Spearman correlation analysis of the risk score and immune cells based on the XCELL, TIMER, QUANTISEQ, MCPCOUNTER, EPIC, CIBERSORT-ABS, and CIBERSORT algorithms.
